# Diagnostic and Prognostic Value of the Triglyceride-Glucose Index in Acute Pancreatitis: A Prospective Observational Study

**DOI:** 10.7759/cureus.111225

**Published:** 2026-06-21

**Authors:** Shubhransu Patro, Preetam Nath, Prithviraj U Naik, Parmarth Arora, Sai sri Karlapudi, Varun Jindal, Mouneesh Karanam, Vibha Sharma

**Affiliations:** 1 General Medicine, Kalinga Institute of Medical Sciences, Bhubaneswar, IND; 2 Gastroenterology, Kalinga Institute of Medical Sciences, Bhubaneswar, IND

**Keywords:** acute pancreatitis, bisap score, diagnostic accuracy analysis, high morbidity, length of hospitalization, modified marshall score, organ dysfunction, prognostic performance, roc curve analysis, triglyceride-glucose index (tyg)

## Abstract

Background and objectives: Acute pancreatitis is a serious, life-threatening illness. Its early prediction can reduce the risk of organ dysfunction, morbidity, mortality, and hospital burden. Though many validated scores are being used in practice in predicting organ dysfunction and mortality among patients with acute pancreatitis, the triglyceride-glucose (TyG) index can also assess mortality risk in the same condition. However, there is a scarcity of studies assessing the predictive value of the TyG index in the Indian subcontinent. Hence, we planned to evaluate the diagnostic and prognostic value of the TyG index among patients with acute pancreatitis.

Methods: This observational study was conducted at Kalinga Institute of Medical Sciences (KIMS), Bhubaneswar, India, from December 15, 2025, to May 15, 2026. We obtained ethical approval prior to the commencement of the study. We assessed the TyG index in patients with acute pancreatitis admitted to our hospital during the study period. We compared those values with existing validated scores, such as the modified Marshall score and the Bedside Index for Severity in Acute Pancreatitis (BISAP) score. We computed the sensitivity, specificity, diagnostic accuracy, and threshold values of the TyG index for diagnosing organ dysfunction in patients with acute pancreatitis and predicting in-hospital mortality of the study participants. R software (version 4.3.2) was used for data analysis.

Results: We analyzed 185 patients with acute pancreatitis in this study. The study population's mean age was 54.8 ± 9.6 years. In our study, 138 (74.6%) participants were males. The mean serum amylase, lipase, triglyceride, and fasting blood glucose values were 553.3 U/L, 1148.6 U/L, 269.1 mg/dL, and 109.4 mg/dL, respectively. The mean TyG index was 8.98 ± 0.76. 24 (13.0%) patients died during their hospital stay. The sensitivity, specificity, diagnostic accuracy, and threshold values of the TyG index in predicting organ dysfunction in patients with acute pancreatitis were 29.6%, 33.3%, 29.7%, and 7.11, respectively. The sensitivity, specificity, diagnostic accuracy, and threshold values of the TyG index in predicting in-hospital mortality were 70.8%, 51.6%, 54.1%, and 6.70, respectively.

Conclusion: We found that the TyG index had lower diagnostic accuracy for predicting organ dysfunction in patients with acute pancreatitis than the modified Marshall score. However, it had better prognostic performance in assessing the mortality risk.

## Introduction

Acute pancreatitis is defined as an acute inflammatory disease of the pancreas [1]. It generally causes acute abdominal pain and various systemic and local complications [1,2]. According to recent studies, the incidence of acute pancreatitis ranges from 30 to 80 per 100,000 persons [3-5]. Over the past two decades, the incidence of acute pancreatitis has increased [3-6].

The 2012 Revised Atlanta Classification uses the modified Marshall scoring system for assessing organ dysfunction in patients with acute pancreatitis [7,8]. It quantifies the risk of organ failure in the respiratory, renal, and cardiovascular systems using basic clinical parameters, such as oxygen requirements, renal function tests, and blood pressure [7-9]. Patients with a modified Marshall score ≥ 2 in any of these organ systems have a high likelihood of severe organ failure and mandate the requirement for escalation of care [7,10].

The Bedside Index for Severity in Acute Pancreatitis (BISAP) score was designed in 2008 for the early assessment of mortality risk of patients with acute pancreatitis [11]. This five-point scoring system comprises five variables: blood urea nitrogen level > 25 mg/dL, impaired mental status, development of systemic inflammatory response syndrome (SIRS), age > 60 years, and presence of a pleural effusion [11,12]. Patients with a BISAP score ≥ 3 generally have a high risk of mortality and morbidity [13].

The triglyceride-glucose (TyG) index, a surrogate biomarker of insulin resistance, has recently emerged as a promising tool for risk stratification in acute pancreatitis [14]. Recent research papers reported a positive correlation between the TyG values and complications of acute pancreatitis, such as acute necrotic collections (ANC), SIRS, and pleural effusion [15-17]. Though these studies underscore the utility of the TyG index as a low-cost biomarker, the Western or East Asian cohorts support it. There remains a paucity of studies in this regard in the Indian context. Establishing the predictive accuracy of the TyG index in Indian patients with acute pancreatitis could facilitate early triage decisions and optimize intensive care unit (ICU) resource allocation.

We aimed to evaluate the diagnostic accuracy of the TyG index compared with the modified Marshall score for predicting organ dysfunction in patients with acute pancreatitis. We also planned to assess the prognostic performance of the TyG index in comparison to the BISAP score in predicting in-hospital mortality among the study participants.

## Materials and methods

We conducted this prospective observational study from December 15, 2025, to May 15, 2026, at Kalinga Institute of Medical Sciences (KIMS), Bhubaneswar, India. The Institutional Ethics Committee (KIIT/KIMS/IEC/2385/2025) gave us ethics approval before we began the study.

Study criteria

The study population comprised all patients admitted to medicine or gastroenterology wards with acute pancreatitis (as per the revised Atlanta classification [9]) during the study period. We included adult patients with acute pancreatitis who presented within five days of the onset of symptoms and stayed in the hospital for > 24 hours. We excluded patients with any kidney, liver, hematological disorder, immunocompromised state, malignancy, or recent blood component transfusion. Pregnant women and patients on either insulin or statin therapy were also excluded.

Study procedure

Eligible patients were enrolled after obtaining written informed consent. Baseline demographics, clinical features, and laboratory data were recorded at admission. Fasting samples at the baseline visit were used to measure serum triglycerides and fasting blood glucose. The TyG index was calculated using the formula: TyG = ln (fasting triglycerides (mg/dL) × fasting blood glucose (mg/dL) / 2) [14-17]. Severity classification was performed according to the Revised Atlanta 2012 criteria, categorizing patients as mild, moderate, or severe acute pancreatitis. Clinical scores, such as the modified Marshall score and BISAP score, were recorded for comparison. The modified Marshall score predicts the risk of organ dysfunction in patients with acute pancreatitis [7-10]. The BISAP score is widely accepted for predicting mortality risk in acute pancreatitis [11-13]. Patients were followed up for their ICU need and outcomes (discharge or in-hospital mortality).

Statistical analysis

We used consecutive sampling in this study. The continuous data were shown as the mean and standard deviation (SD). The categorical data were shown as numbers and percentages. Inferential statistical analyses were limited, but ROC-based diagnostic performance analyses were performed. We calculated the following parameters for the TyG index: sensitivity, specificity, positive and negative predictive values, positive and negative likelihood ratios, and diagnostic accuracy. We plotted the ROC curve and computed the AUC and its 95% confidence interval (CI). We used the R software (version 4.3.2; R Development Core Team, Vienna, Austria) for data analysis [18].

## Results

During the study duration, 237 patients with acute pancreatitis were admitted. Of them, 19 had malignancies, and 33 were on statin therapy. The remaining 185 (78.1%) patients were included in this study. Table 1 shows the demographic and clinical parameters of the participants. The study population’s average age was 54.8 ± 9.6 years, and 138 (74.6%) participants were males. The mean serum amylase, lipase, triglyceride, and fasting blood glucose values were 553.3 U/L, 1148.6 U/L, 269.1 mg/dL, and 109.4 mg/dL, respectively. The average TyG index was 8.98 ± 0.76.

**Table 1:** Demographic and clinical parameters of the participants (n = 185) The continuous data were shown as mean and SD. The categorical data were shown as numbers and percentages. BMI: body mass index; TyG: triglyceride-glucose index

Parameters	Values
Age (years)	54.8 ± 9.6
Male	138 (74.6%)
BMI (kg/m^2^)	24.4 ± 3.7
Diabetes	107 (57.8%)
Hypertension	71 (38.8%)
Alcohol consumption	91 (49.2%)
Smokers	82 (44.3%)
Serum amylase (U/L)	553.3 ± 118.4
Serum lipase (U/L)	1148.6 ± 231.1
Serum triglyceride (mg/dL)	269.1 ± 51.8
Fasting blood glucose (mg/dL)	109.4 ± 32.9
TyG index	8.98 ± 0.76
Duration of hospital stay	22.5 ± 6.1
Pancreatic necrosis	143 (77.3%)
Organ failure	131 (70.8%)
Severe acute pancreatitis	136 (73.5%)
In-hospital mortality	24 (13.0%)

Diagnostic accuracy of the TyG index in predicting organ dysfunction in patients with acute pancreatitis

**Figure 1:**
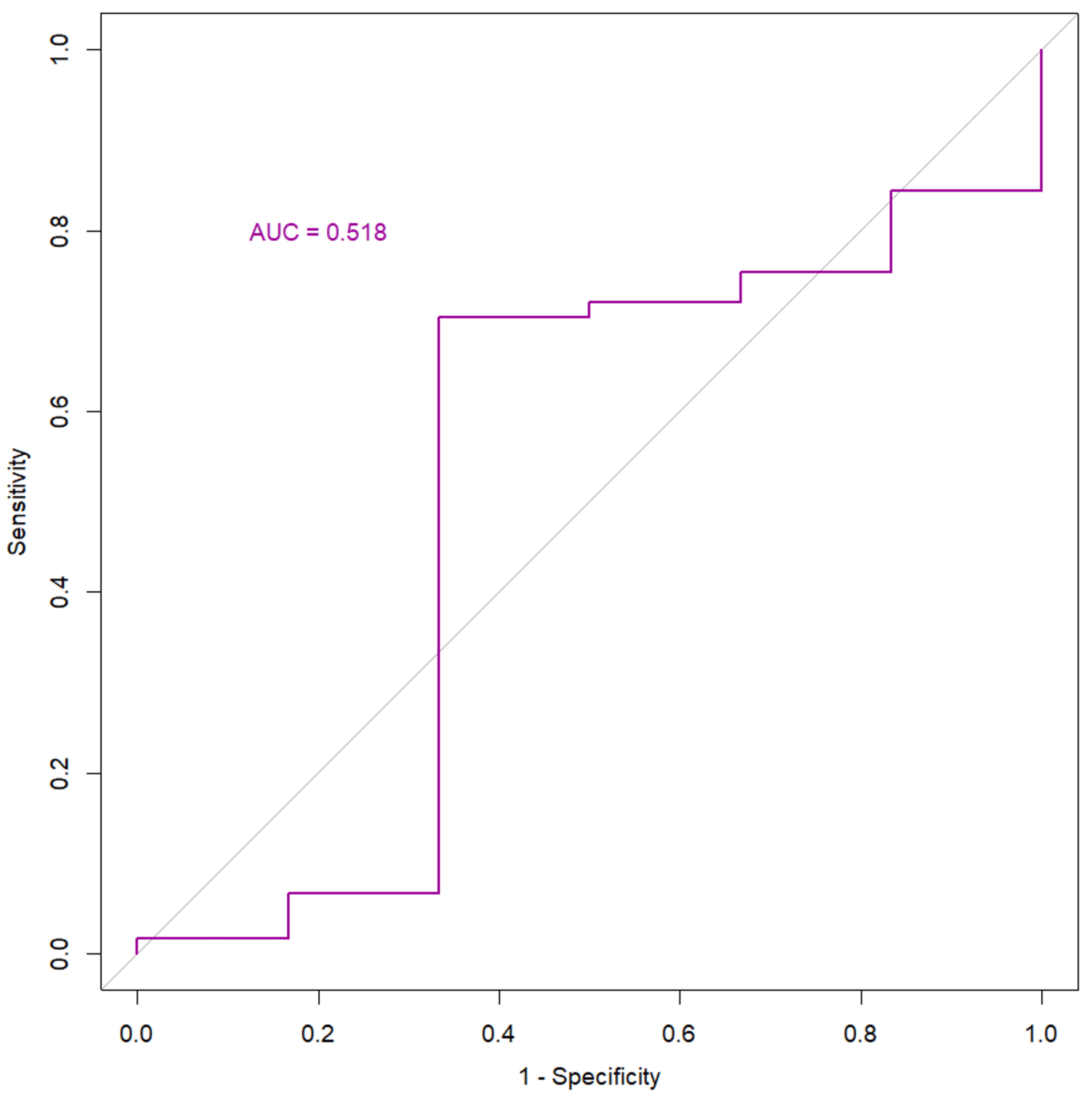
ROC curve for the TyG index in predicting organ dysfunction in patients with acute pancreatitis The X- and Y-axes show 1 - specificity and sensitivity values. ROC curve: receiver operating characteristic curve; TyG index: triglyceride-glucose index; AUC: area under the curve

Tables 2-3 show the contingency table and statistical measures for the TyG index in predicting acute pancreatitis, respectively. The sensitivity, specificity, diagnostic accuracy, and threshold values of the TyG index were 29.6%, 33.3%, 29.7%, and 7.11, respectively. Figure 1 shows the ROC curves of the TyG index in predicting organ dysfunction in patients with acute pancreatitis. The AUC for the TyG index was 0.518 (0.217-0.818).

**Table 2:** Contingency table of the TyG index in predicting organ dysfunction in patients with acute pancreatitis TyG index: triglyceride-glucose index

Parameters	Organ dysfunction	No organ dysfunction	Total
TyG positive	True positive = 53	False positive = 4	57
TyG negative	False negative = 126	True negative = 2	128
Total	179	6	185

**Table 3:** Statistical measures of the TyG index in predicting organ dysfunction in patients with acute pancreatitis TyG index: triglyceride-glucose index; CI: confidence interval

Statistics	Value	95% CI
Sensitivity	29.6%	23.0% to 36.9%
Specificity	33.3%	4.3% to 77.7%
Positive predictive value	93.0%	87.8% to 96.1%
Negative predictive value	1.6%	0.5% to 4.7%
Positive likelihood ratio	0.44	0.24 to 0.82
Negative likelihood ratio	2.11	0.68 to 6.57
Diagnostic accuracy	29.7%	23.3% to 36.9%

Prognostic performance of the TyG index in predicting mortality

**Figure 2:**
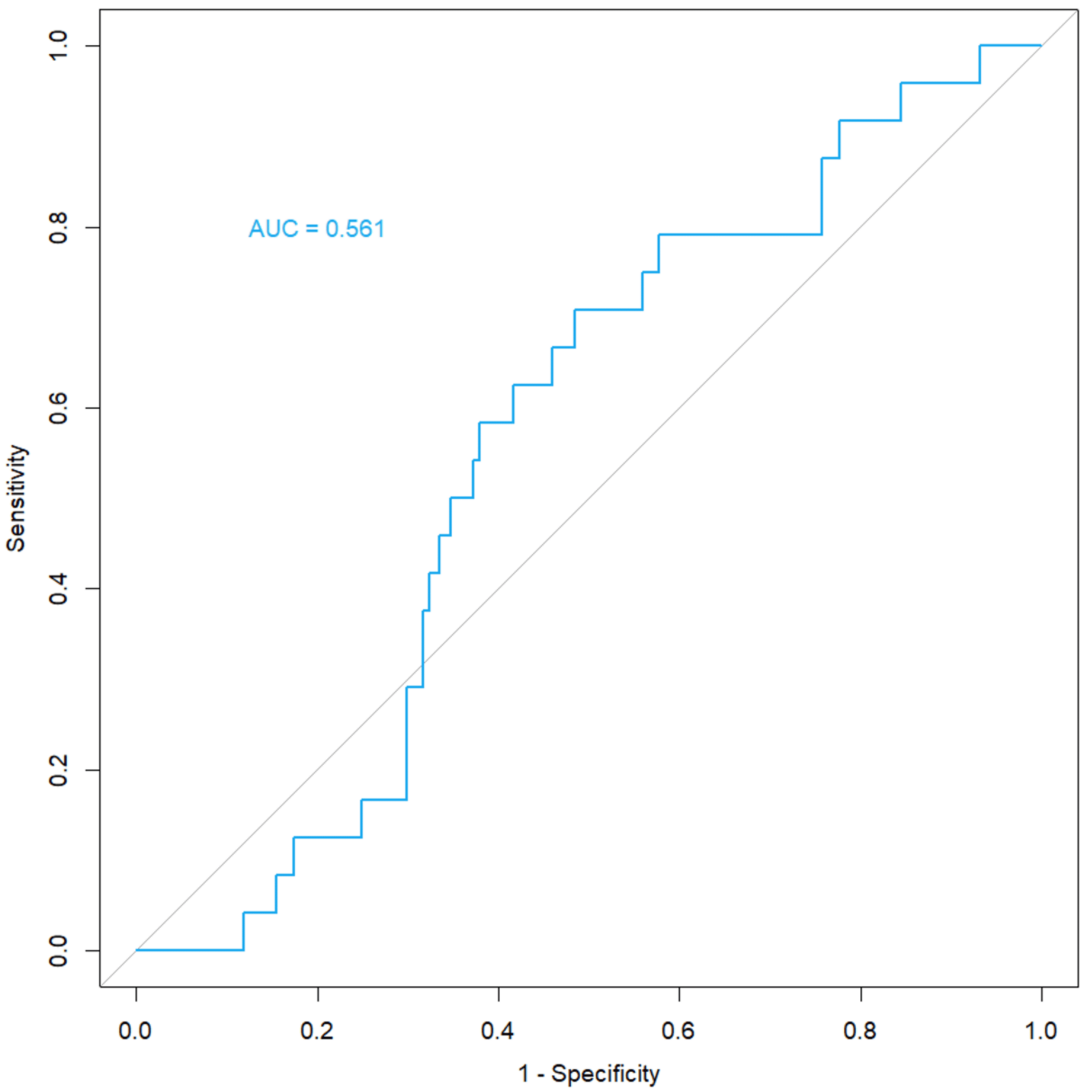
ROC curve for the TyG index in predicting mortality The X- and Y-axes show 1 - specificity and sensitivity values. ROC curve: receiver operating characteristic curve; TyG index: triglyceride-glucose index; AUC: area under the curve

Tables 4-5 show the contingency table and statistical measures for the TyG index in predicting mortality, respectively. The sensitivity, specificity, diagnostic accuracy, and threshold values of the TyG index were 70.8%, 51.6%, 54.1%, and 6.70, respectively. Figure 2 shows the ROC curves of the TyG index in predicting mortality. The AUC for the TyG index was 0.561 (0.455-0.666).

**Table 4:** Contingency table for the TyG index in predicting mortality TyG index: triglyceride-glucose index

Parameters	Mortality	No mortality	Total
TyG positive	True positive = 17	False positive = 78	95
TyG negative	False negative = 7	True negative = 83	90
Total	24	161	185

**Table 5:** Statistical measures of the TyG index in predicting mortality TyG index: triglyceride-glucose index; CI: confidence interval

Statistics	Value	95% CI
Sensitivity	70.8%	48.9%-87.4%
Specificity	51.6%	43.6%-59.5%
Positive predictive value	17.9%	13.9%-22.8%
Negative predictive value	92.2%	86.2%-95.8%
Positive likelihood ratio	1.46	1.08-1.98
Negative likelihood ratio	0.57	0.30-1.07
Diagnostic accuracy	54.1%	46.6%-61.4%

## Discussion

This study underscores the diagnostic accuracy and prognostic performance of the TyG index in patients with acute pancreatitis. We compared the diagnostic accuracy of the TyG index with the modified Marshall score. To assess the prognostic performance of the TyG index, we compared it with the BISAP score. There was a male predominance (138, 74.6%) in our study population. Diabetes and hypertension were the common comorbidities among the study participants. Nearly half of the study population was either an alcoholic and/or a smoker. The demographic findings were consistent with the study by Patel et al. [19]. The mean serum amylase, lipase, triglyceride, and fasting blood glucose values in the study population were 553.3 U/L, 1148.6 U/L, 269.1 mg/dL, and 109.4 mg/dL, respectively. The mean TyG index was 8.98 ± 0.76. Our findings match the recent studies [20-22].

The modified Marshall scoring system quantifies the risk of organ failure in patients with acute pancreatitis [7,8]. Higher scores are suggestive of an increased likelihood of organ dysfunction [7,10]. The BISAP score predicts the risk of morbidity and mortality of patients with acute pancreatitis through a five-point scoring system [11,12]. Higher BISAP scores indicate a worse prognosis in patients with acute pancreatitis [13]. We compared the diagnostic and prognostic values of the TyG index among patients with acute pancreatitis. It is computed from a simple assessment of two commonly used laboratory parameters: serum triglycerides and fasting blood glucose [14].

The sensitivity, specificity, and diagnostic accuracy of the TyG index in predicting organ dysfunction in patients with acute pancreatitis were 29.6%, 33.3%, and 29.7%, respectively. The threshold value of the TyG index in predicting organ dysfunction in patients with acute pancreatitis was 7.11. The AUC for the same was 0.518 (0.217-0.818). The sensitivity, specificity, and diagnostic accuracy of the TyG index in predicting in-hospital mortality among patients with acute pancreatitis were 70.8%, 51.6%, and 54.1%, respectively. The threshold value of the TyG index in predicting in-hospital mortality among patients with acute pancreatitis was 6.70. The AUC for the same was 0.561 (0.455-0.666).

Our study was strengthened by the analysis of two widely used scores, their comparison with the TyG index, and ROC plots. Our study had a few limitations, too. First, the observational design, the smaller sample size, and the short study duration could have affected the statistical performance of the TyG index. Second, we could have compared the diagnostic and prognostic values of the TyG index among patients with chronic pancreatitis. Third, we could not perform any modelling of the TyG index. Our study findings can be generalized through prospective cohort studies with larger sample sizes and longer follow-up periods.

## Conclusions

To conclude, we found that the TyG index had lower diagnostic accuracy for predicting organ dysfunction in patients with acute pancreatitis than the modified Marshall score. However, it had better prognostic performance for mortality. Though it had low diagnostic accuracy in predicting the condition, its prognostic performance for mortality might make it a better tool for primary care physicians for early intervention and timely referral to a higher healthcare facility. Considering the simplicity of this index and its potential to be used in resource-limited regions, we recommend multicentric, prospective studies with larger sample sizes to evaluate the diagnostic and prognostic value of the TyG index in patients with acute pancreatitis.
